# Development of motif-specific monoclonal antibodies for global protein citrullination detection with minimal cross-reactivity to homocitrullination

**DOI:** 10.1016/j.crmeth.2026.101345

**Published:** 2026-03-27

**Authors:** Sophia Laposchan, Erik Riedel, Andrew Flatley, Selina Pasquero, Regina Feederle, Chien-Yun Lee

**Affiliations:** 1 Young Investigator Group: Mass Spectrometry in Systems Neurosciences, School of Life Sciences, Technical University of Munich, 85354 Freising, Bavaria, Germany; 2 Core Facility Monoclonal Antibodies, Helmholtz Zentrum München, German Research Center for Environmental Health, 85764 Neuherberg, Bavaria, Germany; 3 Department of Public Health and Pediatric Sciences, University of Turin, 10126 Turin, Piedmont, Italy; 4 German Center for Neurodegenerative Diseases (DZNE), 81377 Munich, Bavaria, Germany; 5 Munich Cluster for Systems Neurology (SyNergy), 81377 Munich, Bavaria, Germany

**Keywords:** citrullination, monoclonal antibodies, pan-citrullination antibodies, immunodetection

## Abstract

Protein citrullination plays critical roles in biological processes such as immunity, gene regulation, and inflammation. Dysregulated citrullination is implicated in diseases including rheumatoid arthritis, multiple sclerosis, and cancers, making it a potential biomarker and therapeutic target. To overcome current challenges in citrullination detection using immunodetection, we developed motif-based monoclonal antibodies against citrullination. We immunized rats with a pool of over 490,000 citrullinated peptides designed to represent common citrullination motifs in human tissue proteomes. Two monoclonal antibody clones were established and validated for sensitivity and specificity, using ELISA and western blot against *in vitro* citrullinated and homocitrullinated proteomes, as well as ionomycin-activated human neutrophils. Both clones demonstrated great sensitivity to diverse citrullinated proteins, robust discrimination against homocitrullination, and quantitative readout in biological samples. These antibodies provide powerful tools for studying global citrullination dynamics and hold promise for biomarker discovery and diagnostic applications in diseases involving peptidylarginine deiminase (PAD) dysregulation.

## Highlights

• Motif-based immunization with over 490,000 peptides yields pan-citrullination mAbs
• Two mAb clones robustly discriminate citrullination from homocitrullination
• Validated for sensitivity using ELISA, western blot, and immunofluorescence
• The tools enable sensitive, quantitative global profiling of citrullination dynamics

## Motivation

Immunodetection is the most common technique to study citrullination. However, most commercially available antibodies against citrullination are either protein specific or lack the sensitivity and specificity of the broad variety of modified proteins. In addition, existing anti-pan citrullination antibodies often fail to distinguish citrullination from homocitrullination, a chemically similar modification on lysine. This cross-reactivity limits their utility in deciphering the distinct biological roles of these PTMs. To address these challenges, we employed a motif-based strategy to generate monoclonal antibodies against citrullination.

## Graphical abstract

**Figure:**
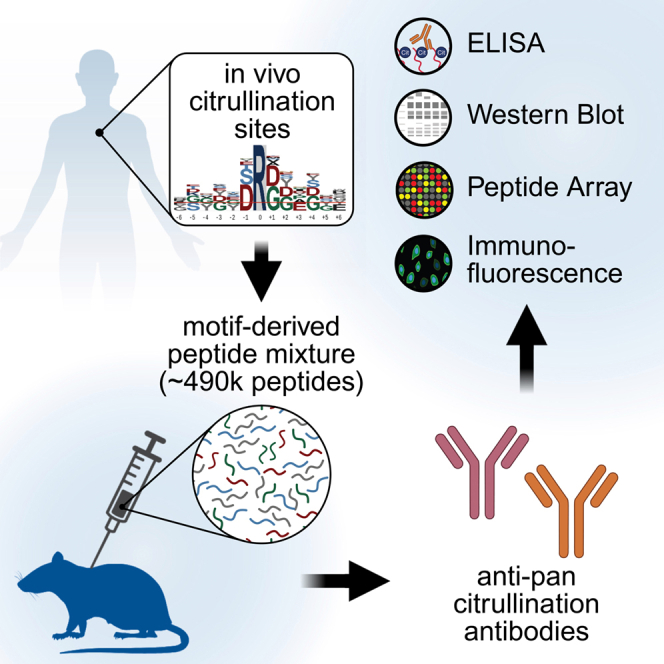

## Teaser

Laposchan et al. report a motif-based strategy to generate monoclonal antibodies for global detection of protein citrullination. The antibodies show strong sensitivity and selectivity over homocitrullination, enabling quantitative detection of global citrullination in cells and lysates. This approach addresses key limitations of existing anti-pan citrullination reagents.

## Introduction

Protein citrullination is a post-translational modification (PTM) catalyzed by peptidylarginine deiminases (PADs) in the presence of calcium, converting arginine residues into citrulline. Its dynamics are critical in diverse biological processes, including innate immunity,^1^ gene regulation,^2^ inflammation,^3^ and apoptosis.^4^ Dysregulation of citrullination is implicated in various diseases, such as rheumatoid arthritis (RA), multiple sclerosis, and cancers.^5^^,^^6^ Detecting citrullination is critical for understanding its cellular functions and the activities of PAD enzymes in both health and disease, and for exploring its potential as a biomarker and therapeutic target. However, current methods for detecting citrullination globally remain limited.

Mass spectrometry (MS) and immunodetection are the primary approaches for PTM analysis, but both face unique challenges in detecting citrullination.^7^ MS, while offering site-specific and quantitative data, is hindered by the small mass change of citrullination (0.98 Da) and the identical mass of deamidation on Gln and Asn. This increases false positives, especially when incorrect monoisotopic precursors are selected or incorrect localization of the 0.98 Da modification occurs on peptides containing Arg, Gln, or Asn.^8^^,^^9^ Although advanced computational strategies^8^^,^^10^^,^^11^ have been proposed to improve data analysis, MS detection requires specialized instrumentation and expertise in sample preparation and data interpretation, limiting its general accessibility.

Compared to MS, immunodetection is widely used due to its accessibility and ease of use. While several anti-citrullination antibodies exist, most are limited to detecting citrullination sites on specific proteins, such as histones and myelin basic protein (MBP) (Table 1). Detecting global changes in citrullination is crucial for understanding PAD enzyme activity and the broader functional roles of citrullination. However, developing sensitive and specific anti-pan citrullination antibodies is challenging due to the subtle structural difference between citrulline and arginine.^7^ The choice of immunogen is, therefore, critical for generating antibodies that can detect small changes in citrullination without being restricted to specific proteins.

**Table 1:** Summary of commercially available protein-specific anti-citrullination antibodies

Supplier	Product number	Target	Host species (isotype)	Clonality [Clone #]	Immunogen	Tested applications^a^
Cell Signaling Technology	40503	histone H3 (cit R2)	rabbit (IgG)	monoclonal [F3C9B]	synthetic peptide of histone H3 with citrullinated R2	WB
Abcam	ab176843	histone H3 (cit R2)	rabbit (IgG)	monoclonal [EPR17703]	not disclosed	I-ELISA, IP, PepArr, and WB
Abcam	ab219406	histone H3 (cit R8)	rabbit (IgG)	monoclonal [EPR20358-13]	not disclosed	ELISA, Flow Cyt (Intra), ICC, IF, IP, and WB
Abcam	ab219407	histone H3 (cit R17)	rabbit (IgG)	monoclonal [EPR20358-120]	not disclosed	ELISA, Flow Cyt (Intra), ICC, IF, IP, and WB
Cell Signaling Technology	97272	histone H3 (cit R17)	rabbit (IgG)	monoclonal [E4O3F]	synthetic peptide of histone H4 with citrullinated R17	IHC and WB
Abcam	ab212082	histone H3 (cit R26)	rabbit (IgG)	monoclonal [EPR20606]	not disclosed	Dot, ELISA, and WB
Abcam	ab281584	histone H3 (cit R2+ cit R8 + cit R17)	rabbit (IgG)	multiclonal [RM1001]	not disclosed	Dot, Flow Cyt (Intra), ICC, IF, IP, PepArr, and WB
Abcam/Bio-Techne	ab5103/NB100-57135	histone H3 (cit R2+ cit R8+ cit R17)	rabbit (IgG)	polyclonal	synthetic peptide of histone H3 amino acids 1–100 (citrulline R2 + R8 + R17) conjugated to KLH	ChIP, ELISA, Flow Cyt (Intra), IF, IHC, IP, PepArr, and WB
Merck/Sigma-Aldrich	07-596	histone H4 (cit R3)	rabbit (IgG)	Polyclonal	synthetic peptide of histone H4 amino acids 1–10 with citrullinated R3	ChIP, ICC, IHC, and WB
Abcam	ab81797	histone H4 (cit R3)	rabbit (IgG)	polyclonal	synthetic peptide of histone H4C1 with citrullinated R3	ELISA, IHC, and WB
Merck/Sigma-Aldrich	MABT1510	MBP (cit R25)	mouse (IgG1)	monoclonal [1B8]	synthetic peptide of isoform 5 of human myelin basic protein with citrullinated R25 (18 amino acids) conjugated to KLH	ELISA, EM, IF, IHC, and WB
Merck/Sigma-Aldrich	MABT1499	MBP (cit R122)	mouse (IgG1κ)	monoclonal [1H1]	synthetic peptide of isoform 5 of human myelin basic protein with citrullinated R122 (19 amino acids) conjugated to KLH	ELISA, IHC, and WB
Merck/Sigma-Aldrich	MABT1488	MBP (cit R130)	mouse (IgG1κ)	monoclonal [3C6]	synthetic peptide of isoform 5 of human myelin basic protein with citrullinated R130 (19 amino acids) conjugated to KLH	ELISA, IHC, and WB
Cell Sciences/Abnova	MON9093/MAB5303	citrullinated fibrinogen	mouse (IgG1)	monoclonal [3D1]	deiminated murine fibrinogen peptide	ELISA, IHC, and WB
Cell Sciences/Biomol	MON9096	citrullinated fibrinogen	mouse (IgG2b)	monoclonal [23H2]	deiminated murine fibrinogen peptide	ELISA, IHC, and WB
Cell Sciences/Biomol	MON9094/USBIF4203-03A	citrullinated fibrinogen	mouse (IgG1)	monoclonal [20B2]	deiminated murine fibrinogen peptide	ELISA, IHC, and WB
Cayman Chemical	34123	citrullinated α-enolase	mouse (IgG1)	monoclonal [4A7]	synthetic citrullinated peptide corresponding to the N-terminal region of human α-enolase	ELISA and WB
Absolute antibody	Ab00224–1.1	citrullinated collagen type II	mouse (IgG1κ)	monoclonal [ACC4]	PAD4-treated helical CII peptides containing T and B cell epitopes emulsified in CFA	ELISA and IHC
Abcam	ab208026	hnRNP A1 (cit R92)	rabbit (IgG)	monoclonal [EPR20174]	not disclosed	Dot, Flow Cyt (Intra), IP, and WB
Abcam	ab208028	hnRNP A1 (cit R97)	rabbit (IgG)	monoclonal [EPR20176]	not disclosed	Dot, IP, and WB
Abcam	ab208029	hnRNP A1 (cit R122)	rabbit (IgG)	monoclonal [EPR20177]	not disclosed	Dot, IP, and WB
Abcam	ab208030	hnRNP A1 (cit R140)	rabbit (IgG)	monoclonal [EPR20178]	not disclosed	Dot and WB
Abcam	ab208027	hnRNP A1 (cit R88+ cit R92)	rabbit (IgG)	monoclonal [EPR20175]	not disclosed	Dot, Flow Cyt (Intra), IP, and WB
Abcam	ab202107	HP1 gamma/CBX3 (cit R108)	rabbit (IgG)	monoclonal [EPR19802-202]	not disclosed	Dot, Flow Cyt (Intra), IP, and WB
Abcam	ab208015	nucleophosmin (cit R196)	rabbit (IgG)	monoclonal [EPR20172]	not disclosed	Dot, IP, and WB

a ChIP, chromatin immunoprecipitation; Dot, dot blot; ELISA, enzyme-linked immunosorbent assay; EM, electron microscopy; flow Cyt (intra), flow cytometry (intracellular); ICC, immunocytochemistry; I-ELISA, indirect ELISA; IF, immunofluorescence; IHC, immunohistochemistry; IP, immunoprecipitation; PepArr, peptide array; WB, western blot.

The earliest approach to address this challenge uses chemically modified citrullinated peptides as an immunogen to enhance immunogenicity, producing anti-modified citrulline (AMC) antibodies.^12^ Despite being highly sensitive, these antibodies require additional derivatization steps on the samples and are only applicable to western blotting and ELISA. Other strategies involve single citrullinated peptides or citrulline-conjugated carrier proteins (e.g., KLH or BSA), but these often suffer from sequence context-dependent recognition, which limits their ability to detect a broad range of citrullinated proteins. Another widely used antibody, F95, was generated using a peptide with 10 citrullines as an immunogen and has demonstrated utility in detecting citrullination in the human brain.^13^ Table 2 summarizes the immunogens and applications of commercially available anti-pan citrullination antibodies.

**Table 2:** Summary of commercially available anti-pan citrullination antibodies

Supplier	Product number	Target	Host species (isotype)	Clonality (clone #)	Immunogen	Tested applications^a^
Merck/Sigma-Aldrich	MABN328	citrulline	mouse (IgMκ)	monoclonal (F95)	citrullinated peptide consisting of 10 citrulline residues and a carboxyl Gly-Gly-Cys	ICC, IHC, and WB
Abcam/Thermo Fisher Scientific/Merck/Sigma-Aldrich	ab240908/MA5-27574/SAB5202292	citrulline	mouse (IgG1)	monoclonal (6C2.1)	synthetic L-citrulline conjugated to KLH	ICC, IF, IHC, ELISA, and WB
Merck/Sigma-Aldrich	SAB5202274/SMC-500	citrulline	mouse (IgG1)	monoclonal (2D3.1)	synthetic L-citrulline conjugated to KLH	ICC, IF, IHC, and WB
Creative Biolabs	HPAB-AP605-YC	citrulline	mouse (IgG)	monoclonal (12G1)	cyclic-structured synthetic peptide, which included a citrullinated filaggrin subunit	ELISA, IHC, and WB
Abcam	ab100932	citrulline	rabbit (IgG)	polyclonal	citrulline coupled to KLH via glutaraldehyde	ELISA and ICC
Merck/Sigma-Aldrich	AB5612	citrulline	rabbit	polyclonal	citrulline-glutaraldehyde-BSA	ELISA and IHC
Merck/Sigma-Aldrich	07-377	citrulline	rabbit (IgG)	polyclonal	linear peptide containing citrulline	IHC and WB
LSBio	LS-C145131	citrulline	rabbit	polyclonal	citrulline coupled to KLH via glutaraldehyde	ELISA, ICC, and WB
Biorbyt	orb10970	L-citrulline	rabbit (IgG)	polyclonal	KLH conjugated to citrulline	ELISA, IF, and IHC
Bioss/Abbiotec	bs-0619R/251244	L-citrulline	rabbit (IgG)	polyclonal	KLH conjugated to citrulline	ELISA and IHC
Merck/Sigma-Aldrich	MABS487	modified citrulline	human (IgG1λ)	monoclonal (C4)	citrulline-containing peptide modified with 2,3-butanedione monoxime and antipyrine	ELISA and WB
Creative Biolabs	ZG-453C	modified citrulline	mouse (IgG2a, κ)	monoclonal (4CS)	citrulline-containing peptide modified with 2,3-butanedione monoxime and antipyrine	ELISA and WB

a ChIP, chromatin immunoprecipitation; Dot, dot blot; ELISA, enzyme-linked immunosorbent assay; EM, electron microscopy; Flow Cyt (Intra), flow cytometry (intracellular); ICC, immunocytochemistry; I-ELISA, indirect ELISA; IF, immunofluorescence; IHC, immunohistochemistry; IP, immunoprecipitation; PepArr, peptide array; WB, western blot.

While anti-citrullinated protein antibodies (ACPAs) from RA patients can develop cross-reactivity to multiple PTMs (e.g., acetylation on lysine),^14^^,^^15^ the antibodies generated for detecting pan-citrullination—such as AMC and F95—often face a different but related challenge: distinguishing between citrullination on arginine and homocitrullination on lysine due to the structural similarity of the modified residues used for immunization.^16^^,^^17^ Homocitrullination, also known as lysine carbamylation, occurs when lysine is chemically modified by cyanate, a potential byproduct of inflammation.^18^ Although both PAD activity and cyanate levels are elevated in inflammatory diseases such as RA, citrullination and homocitrullination are distinct modifications. Discriminating between these PTMs is essential for understanding their respective roles in disease mechanisms.

To address these challenges, we adopted a motif-based approach to generate anti-pan citrullination antibodies. Similar strategies have been used to create pan-PTM antibodies for phosphorylated S/T on the consensus motif of AGC kinase subfamilies (Akt and PKC),^19^ methylarginine GAR motif,^20^ and ubiquitin remnant motif (K-ε-GG).^21^ A key distinction between citrullination and homocitrullination is that citrullination is catalyzed by PADs, which exhibit sequence-specific preferences adjacent to citrullination sites. Antibodies targeting PAD-specific motifs can potentially enhance specificity for citrullination while reducing cross-reactivity with homocitrullination. A similar concept was previously explored using polyclonal antibodies recognizing the R(cit)GG motif,^22^ though sensitivity was limited because this motif accounted for only ∼20% of substrates.

**Figure 1:**
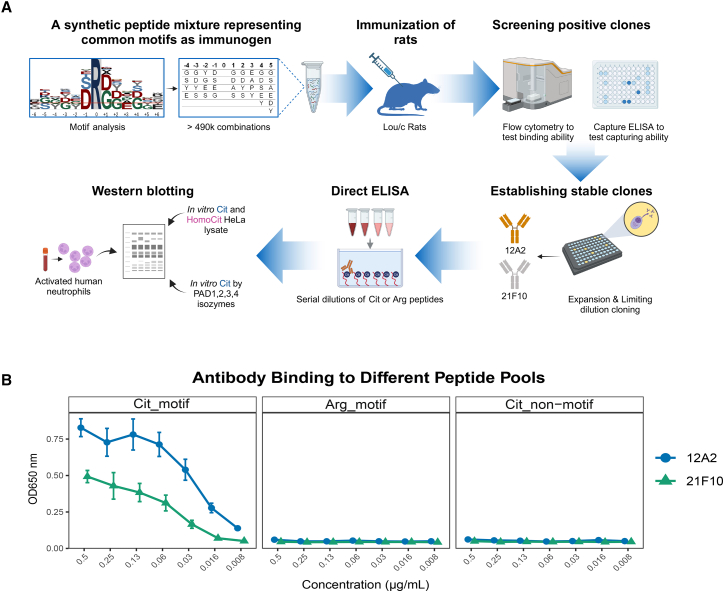
Generation of motif-specific, monoclonal anti-pan citrullination antibodies (A) Schematic overview of the study design. Arg, arginine; Cit, citrulline/citrullination; HomoCit, homocitrulline/homocitrullination. (B) Binding specificity of monoclonal antibodies 12A2 and 21F10 toward different peptide pools. Motif-specific pools (Cit_motif and Arg_motif) comprise peptides with a central citrulline or arginine, respectively, and flanking positions according to the immunization pool. The complementary non-motif pool (Cit_non-motif) contains citrullinated peptides that lack the motif, using only residues absent from the motif pools. Optical density (OD) values are plotted as the mean over triplicate assays, with the error bars showing the standard deviation.

Here, we developed monoclonal antibodies by immunizing with a pool of over 490,000 citrullinated peptides representing common citrullination motifs observed in human proteomes (Figure 1A). We established two stable clones, validated their sensitivity and specificity using direct ELISA as well as western blotting against *in vitro* citrullinated/homocitrullinated proteomes, PAD isozyme-treated citrullinomes, and ionomycin-activated neutrophils. Both clones demonstrated greater sensitivity for citrullination and specificity for citrullination over homocitrullination. These antibodies hold promise for advancing citrullination research and supporting biomarker discovery and diagnostics.

## Results

### Generation of immunization peptide pool

Choosing appropriate immunogens is the key to generating successful antibodies for specific applications. Traditionally, three types of immunogens have been used to produce anti-pan citrullination antibodies: (1) single citrullinated peptide, (2) single amino acid L-citrulline conjugated to carrier proteins like KLH or BSA, and (3) long chains of 10 consecutive citrullines^13^ (Table 2). These approaches share a common limitation: they do not account for the sequence context surrounding the modified site. Because antibodies typically recognize epitopes of 6–12 amino acids in length,^23^^,^^24^ those generated using single peptide, protein, or non-contextual citrulline modifications may lack the broad specificity necessary to detect citrullinated sites with diverse adjacent residues.

Based on our prior identification of PAD2- and PAD4-specific motifs in human tissue proteomes,^9^ we hypothesized that immunogens incorporating common motifs of citrullination sites could enhance specificity and reduce cross-reactivity with homocitrullination. To test this, we first synthesized a pool of diverse 10-residue peptides, incorporating 4–6 different amino acids per position (Table 3). These amino acids were selected based on their statistically significant enrichment (*p* < 0.05; Bonferroni corrected) at corresponding positions in citrullination motifs compared to the human proteome. The percentages of amino acids at each position were weighted according to their observed frequencies, resulting in a pool of over 490,000 unique peptide combinations. This immunogen pool captures the most prevalent citrullination motifs in the human proteome, providing a robust foundation for generating motif-specific antibodies.

**Table 3:** Frequency at each position of the pool of synthetic peptides

-4	−3	−2	−1	0^a^	1	2	3	4	5
G (25%)	G (25%)	G (25%)	D (40%)		G (40%)	G (40%)	E (35%)	G (20%)	G (20%)
S (25%)	D (25%)	S (25%)	S (40%)		D (40%)	D (40%)	A (25%)	D (20%)	D (16%)
Y (25%)	S (25%)	Y (25%)	G (10%)		S (10%)	Y (10%)	P (25%)	Y (20%)	S (16%)
E (25%)	Y (25%)	E (25%)	E (10%)		A (10%)	S (10%)	Y (15%)	S (20%)	Y (16%)
								E (20%)	A (16%)
									E (16%)

a Position of citrulline or arginine.

### Selection of specific monoclonal antibodies against a pool of citrullinated peptides

After immunizing rats with the citrullinated peptide pool, spleen cells from immunized animals were fused with a myeloma cell line to generate hybridoma clones for antibody production. Initial screening was conducted using bead-based flow cytometry (Figure 1A). Two pools of biotinylated peptides—either containing citrulline or arginine—were coupled to streptavidin beads conjugated with distinct fluorophores in the same reaction. Among the screened supernatants from 48× 96-well plates, two (12A2 and 21F10) demonstrated specific binding to citrullinated peptides while showing no interaction with their arginine counterparts, indicating high specificity.

To evaluate the ability of these clones to capture citrullinated peptides, a capture ELISA was performed. Plates were coated with subclass-specific anti-rat antibodies to immobilize the secreted antibodies in the hybridoma supernatants. Biotinylated citrullinated peptides were then added, and binding was detected using HRP-labeled streptavidin. Both clones exhibited significant binding to citrullinated peptides, which confirmed their capability to capture epitopes within the immunogen pool (data not shown).

After limiting dilution cloning and expansion, the binding affinity of the two clones was further tested using a dilution series of biotinylated peptide pools. Three peptide pools were prepared to evaluate specificity toward citrullination and its associated motifs: (1) the motif-specific citrullinated peptide pool used for immunization (Cit_motif); (2) the corresponding motif with citrulline substituted for arginine (Arg_motif); and (3) a non-motif citrullinated peptide pool composed of peptides lacking the preferred residues of the Cit_motif pool (Cit_non_motif). These peptides were immobilized on avidin-coated plates and incubated with hybridoma supernatants. As shown in Figure 1B, both clones demonstrated strong specificity for the motif-specific citrullinated peptide pool, with negligible binding to arginine and non-motif citrulline peptide pools. Notably, clone 12A2 displayed higher signal than clone 21F10, indicating its higher affinity for the targeted motif.

### Epitope mapping using peptide microarrays

Having confirmed general motif-specific binding using pooled peptides, we next sought to investigate the binding preferences of each clone at the individual peptide level. To this end, we performed epitope mapping using a high-density peptide microarray. The array contained 64,480 unique citrullinated 7-mer peptides in triplicates, as well as blank spots to assess baseline signal and non-specific binding. Two peptide groups were designed: (1) Motif group (Cit_motif): peptides containing only preferred amino acids at positions −3 to +3 (except for position +1, where all 20 amino acids were included). (2) Non-motif group (Cit_non_motif): peptides composed of amino acids not present in the Cit_motif group at each position; residues enriched at position +1 (G, S, A, and D) were specifically excluded.

**Figure 2:**
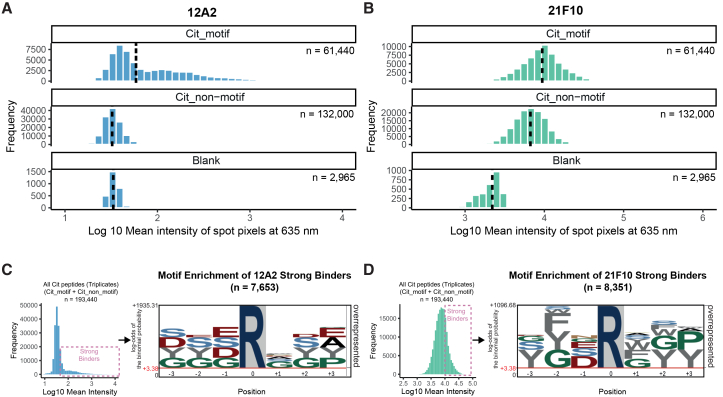
Epitope mapping using high-density peptide microarrays reveals consensus motif for strong binding (A and B) Distribution of binding strength for 12A2 (A) and 21F10 (B) toward motif-specific (Cit_motif, upper panel) and non-motif-specific (Cit_non-motif, middle panel) citrullinated peptides compared to empty spots (blank, lower panel). Vertical dashed lines mark the median of the binding signal. n indicates the numbers of peptide spots. (C and D) Left: Distribution of antibody binding across all tested peptides; the pink dashed box denotes the top quartile used to define “strong binders.” Right: Sequence motif analysis derived from the top-quartile set, with enrichment calculated relative to all tested peptides. n indicates the numbers of unique peptides.

Binding strength was quantified by calculating the mean signal intensity for each spot (Figures 2A and 2B; Table S1). Both antibodies showed significantly higher signal distributions toward the motif group than toward the non-motif group and blank controls, consistent with motif-specific recognition. Notably, clone 12A2 displayed a bimodal signal distribution, indicating selective recognition of a high-affinity subset of motif peptides. In contrast, clone 21F10 exhibited a more uniform distribution across Cit_motif peptides and moderate binding to some non-motif peptides, suggesting broader but still motif-aligned specificity.

To further characterize sequence preferences, we performed motif analysis on the top 25% of binding peptides, regardless of group assignment (*n* = 7,652 for 12A2, and *n* = 8,351 for 21F10) (Figures 2C and 2D). For clone 12A2, this analysis successfully reconstructed the original immunization motif (Figure 2C and Table 3), confirming its high specificity. Clone 21F10 also reflected the general motif architecture, though with slightly broader residue tolerance (Figure 2D). These results further validate the motif-specific nature of both antibodies and suggest that 21F10 may recognize a wider range of citrullinated epitopes while still retaining core motif preferences.

### Reactivity of custom and commercial antibodies toward citrullination and homocitrullination in western blotting

Given the specificity of 12A2 and 21F10 monoclonal antibodies toward citrullinated peptides, we further evaluated their ability to detect a broad range of citrullinated proteins in human proteomes. Proteins extracted from the HeLa cervical cancer cell line were treated overnight with recombinant PAD4 enzyme in the presence of calcium to induce citrullination, or with a high concentration of cyanate to induce homocitrullination. The presence of citrullinated and homocitrullinated proteins was confirmed by MS (Table S2). Notably, the identified citrullination sites aligned with the motif used in the immunogen design, whereas homocitrullination sites did not exhibit any specific motif preference (Figures S1A and S1B). Western blotting was then performed to assess antibody reactivity toward these modifications. For comparison, three commercially available anti-pan citrullination antibodies—07-377, ab100932, and MABN328 (F95)—were included, as their reactivity to citrullinated and homocitrullinated antigens is well characterized.^16^^,^^25^

**Figure 3:**
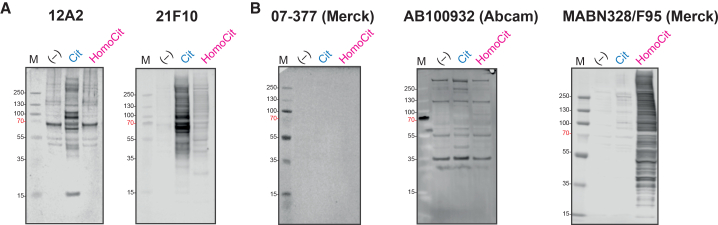
Reactivity of custom and commercial anti-pan citrullination antibodies in western blot analysis of *in vitro* citrullinated (Cit) and homocitrullinated (HomoCit) HeLa cell lysates (A) Reactivity of motif-specific monoclonal antibodies 12A2 and 21F10 against *in vitro* citrullinated or homocitrullinated HeLa proteomes. (−), untreated HeLa lysate. (B) Reactivity of three commercially available anti-pan citrullination antibodies (07-377, ab100932, and MABN328 [F95]) against citrullinated and homocitrullinated HeLa lysates. (−), untreated HeLa lysate.

Both 12A2 and 21F10 specifically detected citrullinated proteins in PAD4-treated HeLa lysates (Figures 3A and S2A), with 21F10 displaying a particularly strong and specific signal and no detectable reactivity in negative controls. While 12A2 showed minimal background signal in the negative control and a faint band in the homocitrullinated sample around 60 kDa, both clones displayed markedly lower signal in homocitrullinated samples than in citrullinated ones, demonstrating great specificity for citrullination.

In contrast, the commercial antibodies exhibited varying performance (Figures 3B and S2B). The 07-377 antibody failed to detect any modified proteins, suggesting low sensitivity. The antibody ab100932 produced non-specific signals, even in negative control samples, aligning with the manufacturer’s recommendation that it can be used exclusively for ELISA and immunocytochemistry (Table 2). MABN328 (F95) showed strong cross-reactivity toward homocitrullination. When probing citrullination and homocitrullination at comparable levels, F95 displayed even greater reactivity toward homocitrullinated proteins (Figure 3B). This observation is consistent with those in prior reports, where Verheul et al.^16^ demonstrated F95’s cross-reactivity with homocitrullinated fibrinogen and albumin, while Chen et al.^25^ noted its non-specific binding to unmodified BSA.

To determine whether the motif-based antibodies could recognize citrullinated proteins catalyzed by different PAD isozymes, we treated HeLa lysates with recombinant PAD1, PAD2, PAD3, and PAD4 enzymes and performed western blotting (Figure S3). Both clones could detect citrullinated proteins in all PAD-treated samples, with slightly higher signal intensity in PAD1-treated lysates. Although no strong isoform-specific reactivity pattern was observed, the sets of proteins recognized by the two clones differed, consistent with their distinct motif preferences.

Together, these results demonstrate that 12A2 and 21F10 selectively recognize PAD-catalyzed citrullination in proteomes and exhibit minimal cross-reactivity toward homocitrullination. Their broad applicability across PAD isoforms makes them valuable tools for studying protein citrullination in diverse biological systems.

### Detection of global citrullination changes during neutrophil activation

The above analyses demonstrate that 12A2 and 21F10 recognize a wide array of citrullinated substrates, with 21F10 showing a higher sensitivity. However, its sensitivity in detecting citrullination in biological samples remains unclear due to the artificially high levels of citrullination in *in vitro* experiments (∼9%–10%). To evaluate the ability of 21F10 to detect (semi-)quantitative changes in global citrullination, we isolated human neutrophils and treated them with the calcium ionophore ionomycin in a dose-dependent manner. This treatment is known to activate PADs, elevate citrullination levels, and induce neutrophil extracellular trap (NET) formation.^26^

**Figure 4:**
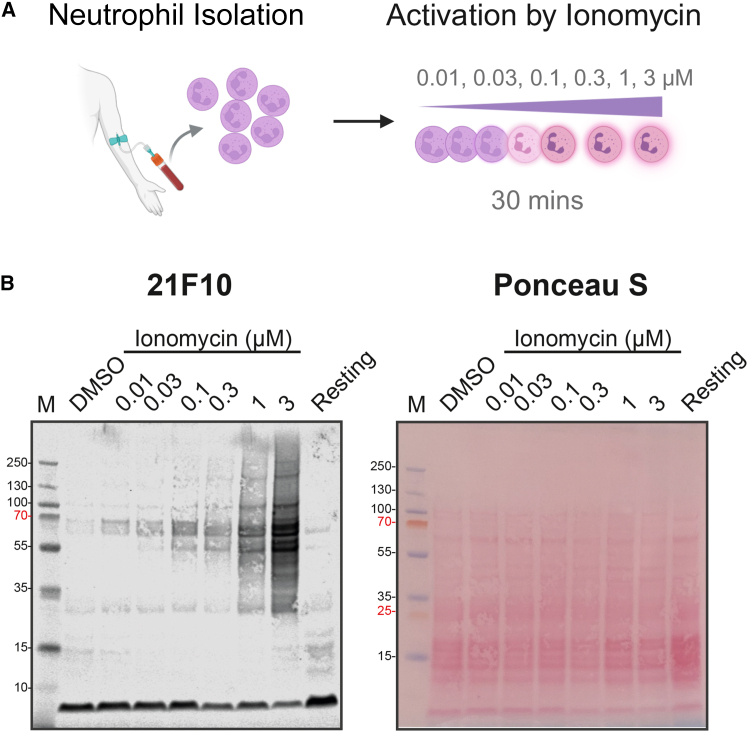
Detection of global citrullination changes during neutrophil activation (A) Schematic of neutrophil activation by ionomycin treatment. (B) Reactivity of the motif-specific, monoclonal antibody 21F10 against citrullinated proteins in ionomycin-activated neutrophil proteomes. Left: citrullination signals detected by western blot. Right: Ponceau S staining confirming equal protein loading across samples.

We detected a dose-dependent increase in signal in the global citrullination levels by 21F10 (Figure 4). These results indicate that 21F10 can effectively recognize changes in pan-citrullination associated with PAD activation in biological samples in a quantitative manner, showing its potential utility for studying dynamic citrullination processes.

## Discussion

Affinity-based detection of target proteins is indispensable in biological research. However, antibody performance varies greatly depending on the target protein and its modifications.^27^ Detecting global level of PTMs via antibodies is particularly challenging, as it requires recognizing a shared epitope independent of the surrounding sequence context. The ubiquitin remnant motif is one successful example, where a motif-specific antibody has been developed to detect ubiquitinated peptides in a sequence-independent manner.^21^

Here, we introduce a new class of anti-pan citrullination antibodies based on motifs preferentially modified by PADs in the human proteome. We assessed their specificity and cross-reactivity within complex biological samples by using western blotting. Through comparative analysis with three commercially available anti-citrullination antibodies, we demonstrated that our custom monoclonal antibodies—particularly clone 21F10—exhibit superior specificity and sensitivity toward citrullinated proteins while displaying minimal cross-reactivity with homocitrullinated proteins. Furthermore, we validated the ability of 21F10 to detect dynamic changes in protein citrullination during neutrophil activation. Although histone-derived signals are commonly observed in activated neutrophils, we did not detect strong bands in the 10–20 kDa range, where core histones typically migrate. This may reflect two factors: first, the motif-guided design of our antibodies, which may limit the recognition of histone citrullination sites that lack the enriched motifs; and second, protocol-specific factors in western blotting, such as membrane pore size and transfer efficiency, which may reduce sensitivity for small proteins. Nonetheless, the antibody effectively captured global citrullination changes following ionomycin-induced PAD activation. Our recent MS-based mapping of the neutrophil citrullinome further supports the notion that citrullination in this context extends well beyond histones.^28^ These findings support the utility of our antibodies as exploratory tools for studying dynamic citrullination in biological systems, rather than as reagents tailored for site-specific detection such as histone modifications, for which validated antibodies already exist (see Table 1).

In addition to evaluations in complex proteomes, we performed high-density peptide microarray analysis to define the epitope preferences of both clones at single peptide level. These results revealed that 12A2 has higher motif specificity for the designated motif used for immunization, whereas 21F10 shows slightly broader residue tolerance while still maintaining general motif preference. This may enable 21F10 to detect citrullinated peptides lacking canonical motifs, which could explain its consistently stronger signal in complex proteomic backgrounds from western blotting. Together, these findings highlight the complementary characteristics of the two clones and suggest that combining motif-specific and motif-tolerant antibodies may enhance both sensitivity and coverage in pan-citrullination detection.

While we evaluated widely used commercial antibodies with well-characterized performance in ELISA (07-377, ab100932, and MABN328) and immunoblotting (MABN328),^16^ we did not cover all reported anti-pan citrullination antibodies. Some lack validation data, while others are not commercially available.^25^ This reflects a broader challenge in the field: the absence of standardized validation protocols for anti-pan citrullination antibodies and the limited accessibility of well-characterized reagents. Establishing reference standards, such as *in vitro* citrullinated lysates or PAD-activated cell models, and ensuring the availability of validated antibodies through reputable research centers or vendors would be instrumental for enhancing reproducibility and broadening accessibility across studies.

In addition to western blotting, we explored the potential of these antibodies in imaging-based detection. In preliminary immunofluorescence experiments using LPS and ionomycin/calcium-activated monocyte cell lines, we observed distinct cell-specific citrullination signals (Figure S4). While further optimization is needed to improve signal specificity and sensitivity, these findings highlight the broader utility of motif-based antibodies for spatially resolved studies of citrullination in cellular systems.

We believe that the continued development of reliable anti-pan citrullination antibodies has the potential to advance fundamental research on citrullination, facilitate biomarker discovery, and improve disease diagnostics. By enabling more accurate detection of citrullinated proteins, these tools may contribute to the identification of novel disease mechanisms and the development of targeted therapeutic strategies.

### Limitations of the study

Although our findings support the feasibility of a motif-based strategy for generating highly specific and sensitive anti-pan citrullination antibodies, certain limitations must be acknowledged. First, although our peptide pool design incorporated over 490,000 motif combinations to comprehensively represent citrullination sites, rare motif variants may not be fully captured, potentially limiting complete citrullinome coverage. Some of these rare motifs—such as those found in known PAD substrates like filaggrin—may be associated with disease and immunogenicity, particularly under conditions of elevated PAD activity. Nevertheless, we envision these antibodies as exploratory tools that, when coupled with MS-based site detection, can help overcome this limitation and provide site-specific information.

Second, despite minimal cross-reactivity with homocitrullinated proteins, the structural similarities between citrulline and homocitrulline residues may still lead to occasional off-target binding. In biological samples with exceptionally high levels of homocitrullination, the resulting signal could obscure citrullination-specific detection. Although such scenarios may be uncommon, integrating MS-based approaches to quantify and distinguish between these modifications would help address this limitation. Notably, MS remains indispensable for precisely characterizing the citrullination sites (+0.9840 Da on Arg) and differentiating them from homocitrullination sites (+43.0058 Da on Lys). Future studies combining high-quality antibodies with advanced proteomic techniques will provide a more comprehensive understanding of citrullination in both physiological and pathological contexts.

Third, in addition to homocitrullination, we acknowledge that broader cross-reactivity with other PTMs, e.g., lysine acetylation, has been reported in RA-derived monoclonal ACPAs.^14^^,^^15^ Although our antibodies were not generated from autoantibody sources and were developed specifically for immunodetection, rather than immune recognition, we cannot fully exclude the possibility of off-target interactions with other modifications. Future work incorporating systematic cross-reactivity testing against a broader panel of modified peptides will help clarify the extent of this risk and further strengthen the utility of motif-based antibodies for citrullination detection.

## Resource availability

### Lead contact

Requests for further information and resources should be directed to and will be fulfilled by the lead contact, Chien-Yun Lee (chienyun.lee@tum.de).

### Materials availability

Both clones, 12A2 (RRID: AB_3677762) and 21F10 (RRID: AB_3677763), generated in this study

are available after payment and upon request from the lead contact and Regina Feederle (regina.feederle@helmholtz-munich.de).

### Data and code availability

• The mass spectrometry proteomics data have been deposited to the ProteomeXchange Consortium via the PRIDE^29^ partner repository with the dataset identifier PXD061971 and are publicly available as of the date of publication.
• The raw peptide array data are included in this paper’s supplemental information (Table S1).
• Original western blot images reported in this paper will be shared by the lead contact upon request.
• This paper does not report original code.
• Any additional information required to reanalyze the data reported in this paper is available from the lead contact upon request.

## Acknowledgments

The authors would like to express their gratitude to all members of the Lee Lab, the Bavarian Center for Biomolecular Mass Spectrometry (BayBioMS), and the Chair of Proteomics and Bioanalytics for their valuable assistance and insightful discussions. This work was funded by the 10.13039/501100002347Federal Ministry of Education and Research, Germany (FKZ031L0215; YIG-SysNS). The Exploris 480 mass spectrometer was funded in part by the 10.13039/501100001659German Research Foundation, Germany (DFG-INST 36/171-1 FUGG). The graphical abstract and illustrations were created with BioRender.com.

## Author contributions

Conceptualization, R.F. and C.-Y.L.; data curation, S.L., E.R., and S.P.; investigation, S.L., E.R., A.F., S.P., R.F., and C.-Y.L.; methodology, S.L., A.F., R.F., and C.-Y.L.; validation, S.L., E.R., and S.P.; writing – original draft, S.L., S.P., and C.-Y.L.; writing – review & editing, S.L., S.P., R.F., and C.-Y.L.; project administration, R.F. and C.-Y.L.; funding acquisition, C.-Y.L.; supervision, C.-Y.L.

## Declaration of interests

The authors declare no competing interests.

## STAR★Methods

### Key resources table

**Table:** 

REAGENT or RESOURCE	SOURCE	IDENTIFIER
**Antibodies**		

Anti-citrullination antibody (clone 12A2)	This paper	RRID: AB_3677762
Anti-citrullination antibody (clone 21F10)	This paper	RRID: AB_3677763
Anti-citrulline polyclonal antibody 07-377	Millipore	Cat# 07-377; RRID: AB_310567
Anti-peptidyl citrulline antibody (clone F95) MABN328	Millipore	Cat# MABN328; RRID: AB_2938608
Anti-citrullination antibody ab100932	Abcam	Cat# ab100932; RRID: AB_10674146

**Biological samples**		

Human peripheral neutrophils	This paper	N/A

**Chemicals, peptides, and recombinant proteins**		

Immunization citrullinated peptide mixture (Cit_motif)	This paper	N/A
Arginine peptide mixture (Arg_motif)	This paper	N/A
Citrullinated peptide mixture, non-motif (Cit_non-motif)	This paper	N/A
Recombinant PAD4	Laboratory of Prof. Hui-Chih Hung, NCHU, Taiwan	N/A
Recombinant PAD1	Biomol	Cat# Cay10784
Recombinant PAD2	Laboratory of Prof. Hui-Chih Hung, NCHU, Taiwan	N/A
Recombinant PAD3	Biomol	Cat# Cay10786

**Critical commercial assays**		

EasySep Direct Human Neutrophil Isolation Kit	STEMCELL Technologies	Cat# 19666

**Deposited data**		

Raw and analyzed mass spectrometry data	This paper	PRIDE, PXD061971

**Experimental models: Cell lines**		

*Mus musculus* Myeloma cell line P3X63-Ag8.653	ATCC	Cat# CRL-1580
Human HeLa cervical cancer cell line	ATCC	Cat# CCL-2
Human THP-1 monocytic cell line	ATCC	Cat# TIB-202

**Experimental models: Organisms/strains**		

Lou/c rats	In-house; first described in Beckers et al.^30^	N/A

**Software and algorithms**		

pLogo motif analysis	O’Shea et al.^31^	https://plogo.uconn.edu/

### Experimental model and study Participant Details

#### Animals

Animal experiments were conducted in accordance with the German animal welfare law and performed with permission and in accordance with all relevant guidelines and regulations of the district government of Upper Bavaria (Bavaria, Germany; Animal protocol number ROB-55.2Vet-2532.Vet_03-17-68). Wildtype Lou/C rats were housed in open cages in groups of 3–4 rats in a 12-h light/dark cycle at 21°C and constant humidity and fed with a standard diet *ad libitum*. Immunization was performed on male and female rats at age between15 and 40 weeks.

#### Cell lines

HeLa cervical carcinoma CCL-2 cells were acquired from ATCC and cultivated in Dulbecco’s Modified Eagle Medium (DMEM) supplemented with 10% FBS, at 37°C and 5% CO_2_. P3X63-Ag8.653 myeloma cells were obtained from ATCC (CRL-1580) and cultured in RPMI 1640 with 10% fetal calf serum, 1% penicillin/streptomycin, 1% glutamine, 1% pyruvate and 1% non-essential amino acids. Human THP-1 monocytic cell line was obtained from ATCC (TIB-202) and cultured in RPMI 1640 medium supplemented with 10% fetal bovine serum, 100 U/mL penicillin, and 100 μg/mL streptomycin, at 37°C with 5% CO_2_.

#### Human

Human neutrophils were isolated from peripheral blood donated by a healthy volunteer (one of the authors). Written informed consent was obtained from the donor. The study was conducted in strict accordance with the Declaration of Helsinki and the guidelines of the Ethics Committee of the Technical University of Munich (TUM) regarding self-experimentation. Specific demographic information (age and sex) is not reported to protect the confidentiality of the single donor.

### Method Details

#### Generation of monoclonal antibodies

A pool of 10aa-long peptides (xxxx-cit-xxxxx; x = mix of 4–6 different aa at each position) against citrullinated motifs identified from human proteome analyses (Table 3) were synthesized and coupled to ovalbumin (Ova) or biotin (Peps4LS, Heidelberg, Germany). Lou/c rats^30^ were immunized subcutaneously (s.c.) and intraperitoneally (i.p.) with a mixture of 40 μg Ova-coupled peptides (∼6–14 mol peptide/mol Ova) in 400 μL PBS, 5 nmol CpG2006 (TIB MOLBIOL, Berlin, Germany), and 400 μL incomplete Freund’s adjuvant. Animals were boosted i.p. and s.c. with the same antigen preparation after 4–7 weeks, and a final boost without Freund’s adjuvant was given 13 weeks after primary immunization. Fusion of the myeloma cell line P3X63-Ag8.653 with the rat immune spleen cells was performed 3 days after the final boost using polyethylene glycol 1500 according to standard procedure.^32^ After fusion, the cells were plated in 96-well plates using RPMI 1640 with 15% fetal calf serum, 1% penicillin/streptomycin, 1% glutamine, 1% pyruvate, 1x non-essential amino acids, 2% HCS (Capricorn) and HAT media supplement (Hybri-Max, Sigma-Aldrich). Hybridoma supernatants from 48 plates were screened 10 days later in a flow cytometry assay (iQue, Sartorius) on biotinylated citrullinated peptides captured on streptavidin beads (PolyAN, Berlin) and incubated for 90 min with hybridoma supernatant and Atto-488-coupled isotype-specific monoclonal mouse-*anti*-rat IgG secondary antibodies (TIB173 IgG2a, TIB174 IgG2b, TIB170 IgG1 all from ATCC, R-2c IgG2c homemade). A pool of biotinylated peptides containing arginine instead of citrulline was used for negative screening. Antibody binding was analyzed using ForeCyt software (Sartorius). Hybridoma cells of clones CIT 12A2 (rat IgG2c) and CIT 21F10 (rat IgG2b) were subcloned by limiting dilution to obtain stable monoclonal cell lines.

#### ELISA assay

Hybridoma supernatants of antibody clones 12A2 and 21F10 were tested in an enzyme-linked immunoassay (ELISA). Binding was measured against pools of biotinylated 10-mer peptides following the scheme described above (Table 3), which contained either citrulline (Cit_motif) or arginine (Arg_motif) in the central position, to assess citrullination specificity. To evaluate motif specificity, we included a third pool (Cit_non-motif) that contained citrulline but no motif. This pool was composed exclusively of residues absent from the motif pools, with equal frequencies at each position. Cysteine and valine were omitted to avoid disulfide bond formation and excessive hydrophobicity. Serial dilutions of peptide (0.5 μg/ml) in PBS with 20% fetal bovine serum (FBS) were added to avidin-coated plates (3ug/ml) overnight. After blocking with 2% FBS in PBS, hybridoma supernatants (1:10 dilution) were added for 30 min. After one wash with PBS, bound antibody was detected with HRP-coupled anti-rat IgG2b (TIB 174, ATCC) or IgG2c antibody (clone 1F8, Helmholtz Munich). HRP was visualized with ready-to-use TMB substrate (1-StepTM Ultra TMB-ELISA, Thermo Fisher) and the absorbance was measured at 650nm with a microplate reader (Spark, Tecan). All ELISA experiments were carried out in triplicates.

#### Peptide microarrays for epitope mapping

High-density peptide microarrays were designed and fabricated in collaboration with axxelera UG (Karlsruhe, Germany). Briefly, two identical 7-mer peptide microarrays, in which every peptide contained a central citrulline (position 0), were constructed. The Cit_motif peptide set comprised all sequence combinations across positions −3 to +3 using the respective amino acids included for these positions in the immunization pool, apart from position +1, where all 20 amino acids were included, yielding 20,480 unique sequences. As a control for motif specificity, the Cit_non-motif set also contained a central citrulline but, at each flanking position, used only residues absent from the Cit_motif residue set at the matching positions. 44,000 sequences were generated by random combination of these residues. Each peptide was flanked with SG (N-terminus) and SGS (C-terminus) linkers to enhance epitope accessibility and was synthesized in triplicate. The library additionally included EDKFVRYVD synthesis controls (2,495 replicates) and empty spots (2,500 replicates) to monitor background and non-specific binding. Peptides were synthesized *in situ* at 20% surface occupancy on a 3D polymer-coated solid support using an Automated Microarray Synthesizer (axxelera) and arranged in an open-chamber format across two identical microarrays with randomized spot placement to minimize spatial bias. After synthesis, peptides underwent N-terminal acetylation and side-chain deprotection. One array was used per antibody clone. Arrays were blocked for 30 min in Rockland Blocking Buffer (MB-070) and pre-stained for 1 h at room temperature with 100 nM goat anti-rat IgG AF647 antibody (10666503, Invitrogen) in assay buffer (TBST +10% (v/v) blocking buffer) to record reference fluorescence. Subsequently, arrays were incubated with test antibodies (12A2 diluted 1:1, 21F10 diluted 1:9 in assay buffer) for 16 h at 4°C, followed by staining with 100 nM anti-rat IgG–AF647. Scanning was performed on an InnoScan 1100AL (Innopsys) using two excitation channels: 532 nm (artifact detection) and 635 nm (secondary anti-rat IgG fluorescence). The 635 nm intensity served as the binding readout. Signal extraction was performed with Mapix (Innopsys), and “F635 Mean” values were used for quantitative analysis. Top 25% peptides with highest intensity values, irrespective of set assignment, were considered “strong binders” and further subjected to motif enrichment analysis.

#### Cell culture

HeLa cells were cultivated in Dulbecco’s Modified Eagle Medium (DMEM) supplemented with 10% FBS. Prior to harvest, cells were washed twice with ice-cold PBS and then lysed with a mild lysis buffer (50 mM Tris/HCl pH 7.5, 5% glycerol, 1.5 mM MgCl_2_, 150 mM NaCl, 1 mM Na_3_VO_4_, 25 mM NaF, 0.8% IGEPAL, 1 mM DTT). Cell lysate was cleared by ultracentrifugation for 1 h at 150,000 xg at 4°C. Protein concentration of the lysate was determined using Pierce BCA Protein Assay Kit (Thermo Fisher Scientific, Rockford, IL, USA) according to the manufacturer’s instructions.

#### *In vitro* protein homocitrullination assay

5 mg of HeLa lysate (5 μg/uL) was diluted with 1.5 mL PBS and 2.5 mL 2M potassium cyanate solution in PBS, resulting in 1M final concentration of potassium cyanate, and incubated overnight at 37°C with shaking at 600 rpm. To remove residual cyanate before further analysis, buffer was exchanged using Amicon Ultra 10 kDA MWCO filters (Merck, Darmstadt, Germany). 5 mL of homocitrullinated lysate were filled up to 15 mL with PBS and centrifuged at 3224 xg until concentrated to ∼1 mL. Five iterations of centrifugation and dilution were performed to reduce the cyanate concentration to <0.01 mM. Protein concentration was determined again using Pierce BCA Protein Assay Kit. The resulting sample was then lyophilized and reconstituted to the desired concentration for further analysis. A control sample was prepared without potassium cyanate in the solution. For western blots, homocitrullinated samples were diluted five times with control sample to obtain a similar level of modification as the citrullinated samples.

#### *In vitro* protein citrullination assay

5 mg of HeLa lysate (5 μg/uL) was diluted with 4 mL PAD buffer (79 mM Tris pH 7.6, 10 mM CaCl_2_, 2.5 mM DTT in final dilution) and incubated with recombinant PAD4 enzyme (kind gift from Prof. Hui-Chih Hung)^33^ at a 1:100 enzyme:protein ratio overnight at 37°C with shaking at 600 rpm. For inactivation of PAD enzyme, samples were subsequently heated for 10 min at 80°C. Buffer exchange and lyophilization was carried out as described above.

For comparison of the citrullination patterns of different PAD isozymes, 200 μg of HeLa lysate (5 μg/μL) was diluted with 160 μL PAD buffer and incubated with recombinant PAD1 (Cay10784, Biomol), PAD3 (Cay10786, Biomol), PAD2, or PAD4 (both kindly provided by Prof. Hui-Chih Hung) enzyme at 1:100 enzyme:protein ratio overnight at 37°C with shaking at 600 rpm. Enzymes were inactivated by heating samples for 10 min at 80°C. A control sample without any PAD was prepared analogously. Aliquots of the samples were lyophilized before western blot analysis.

#### Neutrophils isolation and activation

Peripheral neutrophils were isolated from 25 mL of whole blood collected from a healthy volunteer using the EasySep Direct Human Neutrophil Isolation Kit (STEMCELL Technologies) via immunomagnetic negative selection, following the manufacturer’s protocol. After isolation, the cells were washed with HBSS (Hank’s Balanced Salt Solution) without calcium and magnesium (Gibco, Thermo Fisher Scientific). Five million cells were set aside as resting controls, while the remaining cells were centrifuged and resuspended in HBSS containing calcium and magnesium (Gibco, Thermo Fisher Scientific). For treatment, five million cells per condition were suspended in 1 mL HBSS and 1 μL of ionomycin stock dilutions (Sigma-Aldrich, dissolved in DMSO) were added at final concentrations of 0.01, 0.03, 0.1, 0.3, and 1 μM. Cells were incubated at 37°C for 30 min with gentle shaking. Following treatment, cells were lysed using 2% SDS in 40 mM Tris-HCl (pH 7.6) supplemented with 1X protease inhibitor cocktail (S8820, Sigma-Aldrich). Lysates were heat-inactivated at 90°C for 10 min, then acidified to 1% TFA for DNA hydrolysis and neutralized with 3M Tris buffer. Protein concentrations were determined using the BCA assay according to the manufacturer’s protocol. Twenty micrograms of protein from each sample were used for western blot analysis.

#### Western blotting

Lyophilized samples (20–30 μg) were reconstituted in 30 μL of NuPAGE LDS sample buffer (Invitrogen) supplemented with 25 mM DTT. Samples were heated at 90°C for 10 min before loading onto NuPAGE 4–12% Bis-Tris gels (10- or 12-well, Invitrogen). PageRuler Plus Prestained Protein Ladder (Thermo Fisher Scientific) was used as a molecular weight marker, and NuPAGE MOPS buffer (Invitrogen) as the running buffer. Electrophoresis was carried out at 200 V for 45 min using an XCell SureLock Mini Cell chamber (Invitrogen). Proteins were transferred onto a 0.45 μm PVDF membrane (Merck Millipore) using NuPAGE transfer buffer (Invitrogen) and the XCell II blot module (Invitrogen) at 30 V for 1 h. Membranes were stained with Ponceau S (Sigma-Aldrich) for 10 min to confirm equal loading and then destained with ultrapure water. Prior to antibody probing, membranes were washed three times with TBST and blocked with 2% BSA in TBST for 1 h at room temperature. Membranes were incubated overnight at 4°C with primary antibodies under gentle shaking using 1) Custom antibodies: 12A2 and 21F10 (unpurified supernatant, 1:10); 2) Commercial antibodies: 07-377 (EMD Millipore, 1:1,000), MABN328 (EMD Millipore, 1:500), and ab100932 (Abcam, 1:500). Following three TBST washes, membranes were incubated with secondary antibodies (LI-COR, Inc.) for 30 min at room temperature: IRDye 800CW-coupled goat anti-rat IgG (926–32219); IRDye 800CW-coupled goat anti-rabbit IgG (926–32211); IRDye 680RD-coupled goat anti-mouse IgM (925–68180). All secondary antibodies were diluted 1:10,000 in TBST. After thorough washing with TBS, fluorescence detection was performed using a Li-Cor Odyssey Scanner (LI-COR, Inc.).

#### Mass spectrometry analysis

Sample preparation: To confirm modifications, 100 μg of control, citrullinated, and homocitrullinated HeLa lysates were reconstituted in digestion buffer (100 mM HEPES, pH 8.5, 10 mM TCEP, 55 mM CAA) and incubated at 37°C for 1 h with shaking for protein reduction and alkylation. Trypsin was added (1:50 enzyme:protein ratio), and samples were digested overnight at 37°C. Digests were acidified with 1% of formic acid and desalted using Sep-Pak C18 SPE cartridges (Waters). Peptides were eluted, lyophilized, and stored at −20°C until MS analysis.

Data acquisition: Samples were reconstituted in 10 μL of 0.1% FA and analyzed via LC-MS/MS on a Vanquish *Neo* LC system coupled to an Orbitrap Exploris 480 mass spectrometer (Thermo Fisher Scientific). A 5 μL injection (ca. 25 μg peptide) was loaded at 100 μL/min onto an Acclaim PepMap RSLC C18 column (Thermo Fisher Scientific), separated using a 60-min gradient (mobile phases A: 0.1% FA, 3% DMSO in H_2_O; B: 0.1% FA, 3% DMSO in ACN) at 50 μL/min, and ionized at 3500 V. The MS was operated in data-dependent acquisition mode with a 1.2 s cycle time, MS1 resolution of 60,000 (m/z 360–1300), and MS2 resolution of 15,000, using HCD fragmentation (28% collision energy) and dynamic exclusion (30 s, 10 ppm tolerance).

Database search and data analysis: Raw data were analyzed using FragPipe (v21.1) (MSFragger v4.0) against a human canonical protein database (Uniprot, 20,409 entries, downloaded 20.11.2023). Trypsin was set as the digestion enzyme (up to 3 missed cleavages, peptide length 7–50 amino acids, mass range 500–5000 Da). Fixed modification: carbamidomethylation (C); variable modifications: oxidation (M), acetylation (N-term), citrullination (R, +0.9840 Da), deamidation (N/Q, +0.9840 Da), and carbamylation (K, +43.0058 Da), allowing up to three modifications per peptide. Rescoring using MSBooster was disabled. All other search parameters were kept at default values. Modification levels were assessed by comparing peptide numbers and intensities. In the citrullinated sample, 21.5% of R-containing peptides and 9.1% of total peptides were modified (11.5% and 3.8% of intensity, respectively). The homocitrullinated sample showed near-complete lysine conversion, with 97.3% of K-containing peptides and 52.8% of total peptides modified (96.1% and 56.1% intensity-based). In contrast, modifications were detected in <0.1% of peptides in the control sample, within the 1% false discovery rate at PSM level.

#### Motif analysis

Potential substrate motifs around citrullination and homocitrullination sites were analyzed using pLogo.^31^ The immunogen peptide pool was designed based on motif analysis of citrullination sites identified in our previous study across 30 human tissues.^9^ Flanking positions −4 to +5 relative to the citrullination site were evaluated, and amino acids that were statistically overrepresented compared to the human proteome (*p* = 0.05, Bonferroni corrected) were included in the peptide mixture at frequencies determined by the motif analysis.

For sequence motif analysis of the *in vitro-*generated citrullination and homocitrullination datasets from this study, sequences spanning five residues upstream and downstream of citrullination (R) or homocitrullination (K) sites were extracted and compared to background frequencies in the human proteomes. Statistically significant motifs (*p* = 0.05, Bonferroni corrected) were visualized.

#### Immunofluorescence assay

Human THP-1 monocytic cell line was obtained from ATCC (TIB-202) and cultured in RPMI 1640 medium (Sigma-Aldrich) supplemented with 10% fetal bovine serum (*Neo* FBS 2.0, Neobiotech), 100 U/mL penicillin, and 100 μg/mL streptomycin, in a humidified incubator at 37°C with 5% CO_2_. To differentiate THP-1 monocytes into macrophages,^34^ cells were seeded in 6-well plates (1.5 × 10^6^ cells per well) or 24-well plates (3 × 10^5^ cells per well) containing sterile coverslips, and treated with 100 ng/mL phorbol 12-myristate 13-acetate (PMA, Sigma Aldrich) for 48 h. After a 3-day resting period, THP-1-derived macrophages were primed with 1 μg/mL lipopolysaccharide (LPS from Escherichia coli serotype o111:b4, Sigma-Aldrich) for 3 h. Citrullination was then induced by treatment with 3 μM ionomycin (Sigma-Aldrich, dissolved in DMSO) and 1 mM CaCl_2_ in serum-free RPMI for 30 min. Control THP-1 macrophages were incubated in serum-free RPMI alone. Following treatments, cells were fixed with 4% paraformaldehyde (PFA, Sigma-Aldrich) for 20 min at room temperature and processed for immunofluorescence analysis.

After fixation with 4% paraformaldehyde (PFA) for 20 min at room temperature, cells were permeabilized with 0.1% Triton X-100 in PBS for 20 min at 4°C, and washed three times for 5 min in PBS. Non-specific binding was blocked with 5% BSA (Sigma-Aldrich) and 2% human serum (Sigma-Aldrich) in PBS for 1 h at room temperature. Cells were then incubated with primary antibodies 21F10 and 12A2 (1:1) diluted in blocking buffer for 2 h at room temperature. After three washes in PBS (5 min each), cells were incubated with Alexa Fluor 568-conjugated goat anti-rat IgG secondary antibody (1:200; A-11077, Invitrogen) for 2 h at room temperature in the dark. Nuclei were counterstained with DAPI (Sigma) for 10 min, followed by two PBS washes (5 min each) and one rinse in sterile water. Coverslips were mounted onto pre-cleaned slides (Gold Seal Rite-On) using 6 μL Vectashield antifade mounting medium (Vector Laboratories). Images were acquired using an Olympus IX70 fluorescence microscope.

### Quantification and statistical analysis

ELISA experiments visualized in Figure 1B were performed as triplicate experiments, and the values depicted represent the mean across triplicates, with error bars showing the standard deviation (also see Figure legend and Method Details).

Motif analysis of modification sites (Figure 2 C,D; Supplemental Figure S1) was performed using pLogo,^31^ which assesses residue enrichment by binomial testing against the human proteome as background with Bonferroni correction for multiple testing. Details on the input and selected parameters are described in Method Details. For more information on the enrichment tool refer to O’Shea et al.^31^

Peptide array data was processes using Mapix (Innopsys), and “F635 Mean” values were used for quantitative analysis. Details (e.g., n numbers, median) can be found in the corresponding Method Details section and legend of Figure 2.

## Supplemental information

Document S1. Figures S1–S4

Table S1. Readout of peptide microarrays performed on antibody clones 12A2 and 21F10, related to Figure 2

Table S2. List of modified peptides detected in *in vitro* citrullinated, homocitrullinated, or control HeLa sample, related to Figure 3

Document S2. Article plus supplemental information
