# Supplementary material for: Development of motif-specific monoclonal antibodies for global protein citrullination detection with minimal cross-reactivity to homocitrullination
Source: Cell Rep Methods. 2026 Mar 27;6(4):101345. doi: 10.1016/j.crmeth.2026.101345 (PMC13106979; doi:10.1016/j.crmeth.2026.101345)
Supplement: Document S1. Figures S1–S4 [file mmc1.pdf]

Cell Reports Methods, Volume *6*

Supplemental information

Development of motif-specific monoclonal antibodies for global protein citrullination detection with minimal cross-reactivity to homocitrullination

Sophia Laposchan, Erik Riedel, Andrew Flatley, Selina Pasquero, Regina Feederle, and Chien-Yun Lee

Supplemental Figure S1

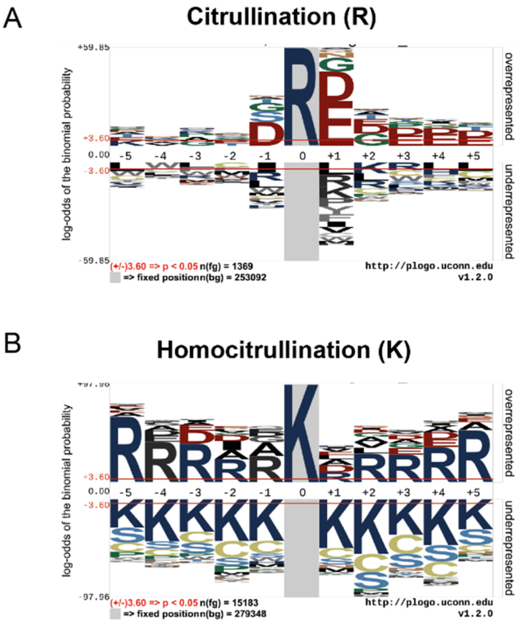

Supplemental Figure S1. Motif analysis of citrullinated and homocitrullinated peptides identified in HeLa lysates, related to Figure 3.

(A) Sequence motif analysis of citrullination sites. (B) Sequence motif analysis of homocitrullination sites.

Supplemental Figure S2

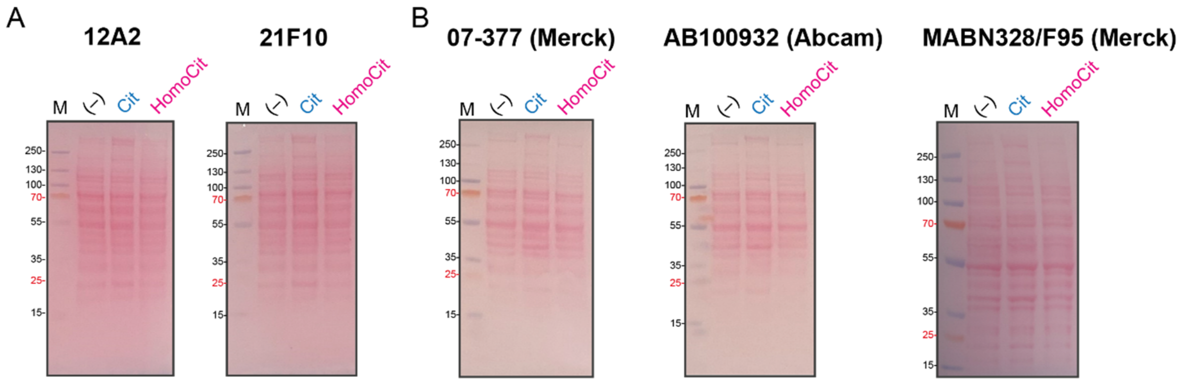

Supplemental Figure S2. Ponceau S staining, related to Figure 3. The plot is corresponding to the blots shown in Figure 3, confirming equal protein loading.

Supplemental Figure S3

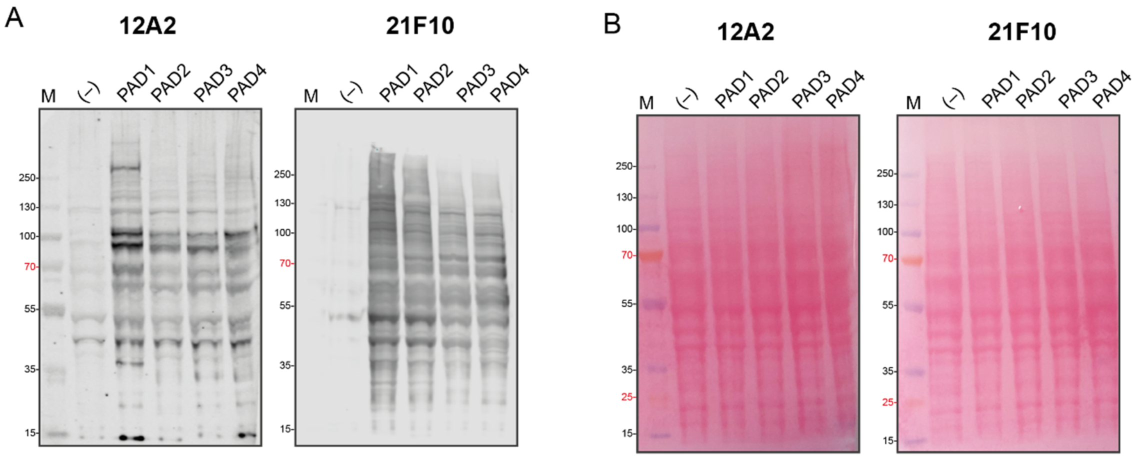

Supplemental Figure S3. Reactivity of custom antibodies in western blot analysis of HeLa lysates citrullinated by different PAD isozymes, related to Figure 3. (A) Reactivity of motif-specific monoclonal antibodies 12A2 and 21F10 toward HeLa proteomes treated with different PAD isozymes (PAD1, PAD2, PAD3, PAD4). (-), untreated HeLa lysate. (B) Ponceau S staining confirming equal protein loading across samples.

Supplemental Figure S4

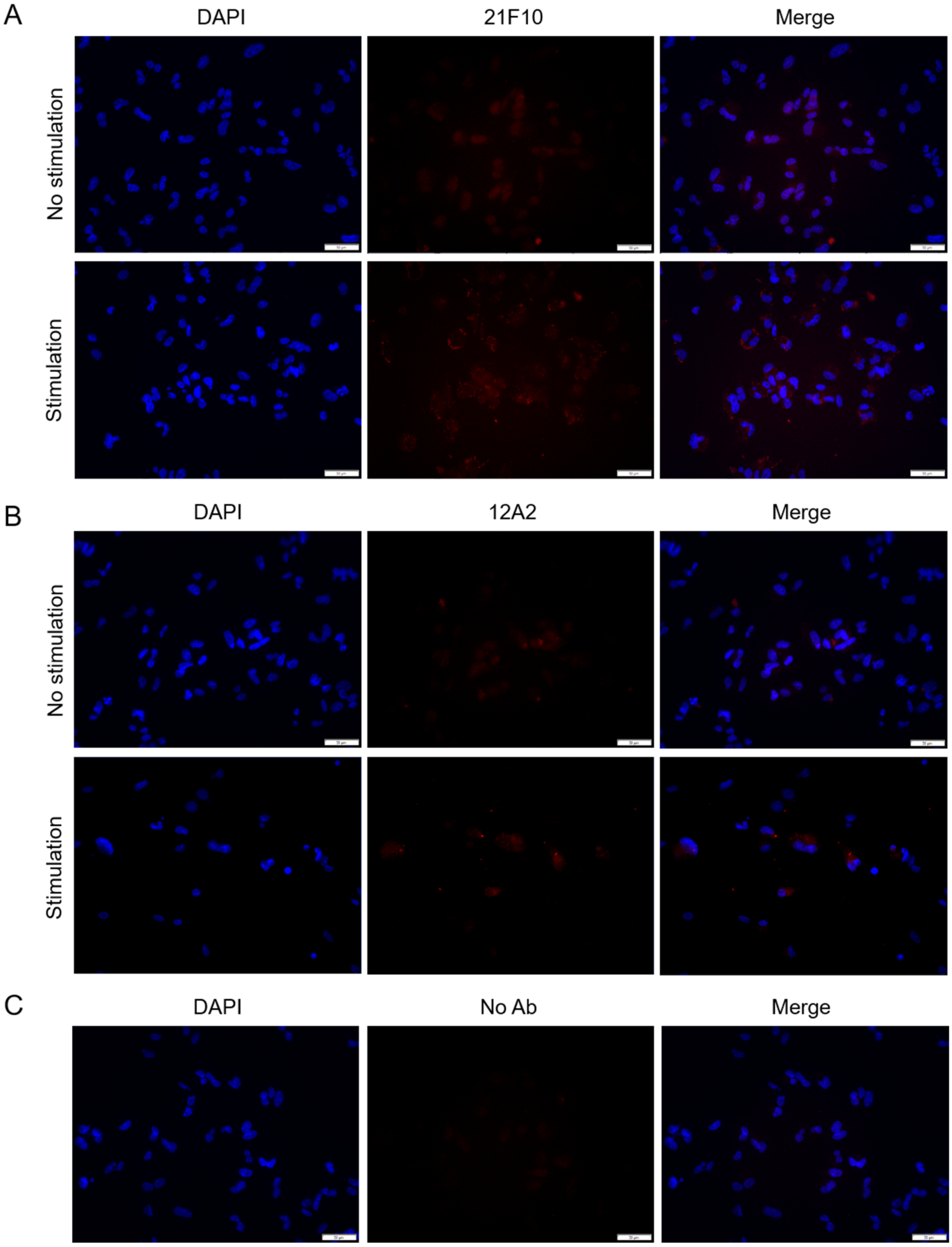

Supplemental Figure S4. Reactivity of custom antibodies in immunofluorescence analysis of cells, related to STAR Methods. (A) Reactivity of the motif-specific monoclonal antibody 21F10 in Thp1 monocyte cells differentiated into macrophages by PMA treatment and either stimulated with ionomycin and calcium or left unstimulated as control (No stimulation). DAPI = blue, 21F10 = red. (B) Reactivity of the motif-specific monoclonal antibody 12A2 in Thp1 cells differentiated into macrophages by PMA treatment and either stimulated with ionomycin and calcium or left unstimulated as control (No stimulation). DAPI = blue, 12A2 = red. (C) Control immunofluorescence performed on Thp1 cells stimulated with ionomycin and LPS in the absence of the primary antibodies 21F10 or 12A2, using only the fluorescent anti-rat secondary antibody. This confirms that the signal observed in panels (A–B) is specific and not due to cross-reactivity of the secondary antibody.
